# Genotypic and Phenotypic Characterization of P23H Line 1 Rat Model

**DOI:** 10.1371/journal.pone.0127319

**Published:** 2015-05-26

**Authors:** Elise Orhan, Deniz Dalkara, Marion Neuillé, Christophe Lechauve, Christelle Michiels, Serge Picaud, Thierry Léveillard, José-Alain Sahel, Muna I. Naash, Matthew M. Lavail, Christina Zeitz, Isabelle Audo

**Affiliations:** 1 INSERM, U968, Paris, France; 2 CNRS, UMR_7210, Paris, France; 3 Sorbonne Universités, UPMC Univ Paris 06, UMR_S 968, Institut de la Vision, Paris, France; 4 Centre Hospitalier National d’Ophtalmologie des Quinze-Vingts, DHU ViewMaintain, INSERM-DHOS CIC 1423, Paris, France; 5 Institute of Ophthalmology, University College of London, London, United Kingdom; 6 Fondation Ophtalmologique Adolphe de Rothschild, Paris, France; 7 Academie des Sciences, Institut de France, Paris, France; 8 Department of Cell Biology, University of Oklahoma Health Sciences Center, Oklahoma City, Oklahoma, United States of America; 9 Department of Ophthalmology, University of California San Francisco, San Francisco, California, United States of America; 10 Department of Anatomy, University of California San Francisco, San Francisco, California United States of America; Justus-Liebig-University Giessen, GERMANY

## Abstract

Rod-cone dystrophy, also known as retinitis pigmentosa (RP), is the most common inherited degenerative photoreceptor disease, for which no therapy is currently available. The P23H rat is one of the most commonly used autosomal dominant RP models. It has been created by incorporation of a mutated mouse rhodopsin (*Rho*) transgene in the wild-type (WT) Sprague Dawley rat. Detailed genetic characterization of this transgenic animal has however never been fully reported. Here we filled this knowledge gap on P23H Line 1 rat (P23H-1) and provide additional phenotypic information applying non-invasive and state-of-the-art *in vivo* techniques that are relevant for preclinical therapeutic evaluations. Transgene sequence was analyzed by Sanger sequencing. Using quantitative PCR, transgene copy number was calculated and its expression measured in retinal tissue. Full field electroretinography (ERG) and spectral domain optical coherence tomography (SD-OCT) were performed at 1-, 2-, 3- and 6-months of age. Sanger sequencing revealed that P23H-1 rat carries the mutated mouse genomic *Rho* sequence from the promoter to the 3’ UTR. Transgene copy numbers were estimated at 9 and 18 copies in the hemizygous and homozygous rats respectively. In 1-month-old hemizygous P23H-1 rats, transgene expression represented 43% of all *Rho* expressed alleles. ERG showed a progressive rod-cone dysfunction peaking at 6 months-of-age. SD-OCT confirmed a progressive thinning of the photoreceptor cell layer leading to the disappearance of the outer retina by 6 months with additional morphological changes in the inner retinal cell layers in hemizygous P23H-1 rats. These results provide precise genotypic information of the P23H-1 rat with additional phenotypic characterization that will serve basis for therapeutic interventions, especially for those aiming at gene editing.

## Introduction

Rod-cone dystrophy, also known as retinitis pigmentosa (RP), is a clinically and genetically heterogeneous group of progressive inherited retinal disorders, which often start with night blindness and lead to visual field constriction, secondary macular involvement and in many cases results in loss of central vision and complete blindness [1]. RP occurs in 1 of 4,000 births and affects more than 1 million individuals worldwide [1]. The mode of inheritance can be autosomal dominant (ad), autosomal recessive (ar), or X-linked. Currently, there is no treatment for this disease but various therapeutic strategies are under investigation.

Mutations in the rhodopsin-encoding gene (*RHO*; MIM# 180380) were the first molecular defects identified in RP [2–5]. More than 100 different *RHO* mutations have been reported to date, which account for 30–40% of autosomal dominant RP (adRP) cases in United States [1] and 16% in France [6]. Furthermore, mutations in 25 other genes cause adRP (http://www.sph.uth.tmc.edu/Retnet/, last accessed date on December 16^th^, 2014) with mutations in *RHO* being the most prevalent [6, 7]. *RHO* encodes for the rod-specific protein rhodopsin, which belongs to the superfamily of 7 transmembrane G-protein—coupled receptors. Rhodopsin consists of an apoprotein (opsin) that is covalently bound to a small conjugated chromophore (11-*cis-*retinal), derived from vitamin A. Upon light absorption, the chromophore isomerizes into all-*trans*-retinal, with subsequent rhodopsin conformational changes leading to the activation of the phototransduction cascade [8].

The P23H exchange in RHO is the most prevalent cause of RP in the United States [3]. In this country, this mutation alone accounts for about 12% of adRP cases and about a third of those with a dominant *RHO* mutation [9]. Previous studies have suggested that the P23H mutant protein is misfolded, retained in the endoplasmic reticulum (ER) and unable to bind 11-*cis*-retinal [10–12]. The misfolded RHO can be degraded through the ubiquitin-proteasome system or aggregates in the cytoplasm [13, 14]. In order to better understand the disease and its pathogenic mechanisms, P23H transgenic albino rats were generated by incorporating a C57BL/6J mouse P23H mutated transgene into a Sprague-Dawley wild-type (WT) rat background. The transgene used was cloned from C57BL/6J mice. It was made from the entire wild type 15kb of the mouse rhodopsin genomic fragment (called lambda 11) as indicated in Al-Ubaidi *et al*. (1990) [15]. The 15 kb genomic fragment lacking the lambda arms with the P23H mutation was used to generate the rat models. It was initially generated to create a mouse model and therefore few variants were inserted in order to screen the mouse transgene in the presence of the wild-type mouse copy. Three lines of P23H transgenic albino rats were generated with this transgene: one represents a fast degeneration (Line 1), one a slower degeneration (Line 3) and one a very slow degeneration model (Line 2). All three lines suffer from a progressive rod degeneration initially associated with normal cone function [16] (UCSF School of Medicine, http://www.ucsfeye.net/mlavailRDratmodels.shtml). These phenotypes are consistent with the clinical findings in patients carrying the P23H *RHO* mutation. Photoreceptor loss correlates with full field electroretinogram (ERG) abnormalities in dark adapted (i.e., scotopic) conditions. Under these conditions, the a-wave amplitude, reflects rod photoreceptor hyperpolarization upon light stimulation, whereas the b-wave represents the subsequent bipolar cell depolarization upon signal transmission from the photoreceptors. Both a- and b- waves of the P23H rat are dramatically reduced over time. In light adapted (i.e., photopic) conditions, the b-wave is normal until the outer nuclear layer (ONL), containing photoreceptor nuclei, thins beyond 50% [17]. In addition to photoreceptor degeneration, there is a subsequent reduction in rod bipolar dendrites [18] and a substantial loss of retinal ganglion cells [19, 20]. By reproducing the clinical signs observed in patients, the P23H rat model is very valuable for testing therapeutic approaches for adRP. Nevertheless, the genotype of this popular animal model has not been fully reported thus far. In this study, we focused on the P23H Line 1 (P23H-1) rat model frequently used due to its fast and sequential degeneration affecting first rod and then cone photoreceptors [17]. We established the exact sequence of the mutated transgene and report copy number and corresponding expression level. In addition, we sought to more precisely document the kinetics of retinal abnormalities applying two *in vivo* techniques, ERG and spectral-domain optical coherence tomography (SD-OCT), a high resolution imaging technique, allowing monitoring the thickness of retinal layers. Both of these *in vivo* assessment techniques are routinely applied to patients with retinal disease.

## Material and Methods

### Animals

Transgenic homozygous P23H-1 rats were obtained from the laboratory of Matthew LaVail (UCSF School of Medicine, http://www.ucsfeye.net/mlavailRDratmodels.shtml) and were crossed with WT albino Sprague-Dawley rats purchased from Janvier (Le Genest-Saint-Isle, France) to produce hemizygous P23H-1 rats. Animals were housed with a 12-hour dark/light cycle with food (standard diet R0425, Scientific Animal Food & Engineering, Augy, France) and water available *ad libitum*.

Light level was measured in the cage with a luxmeter (Illuminance Meter T10, Konica Minolta, Osaka, Japan) and varied between 20 and 180 lux depending on the position of the rat in the cage and the position of the cage in the rack. For more consistence, WT and hemizygous P23H-1 rats of a same group were housed on the same row.

### Ethic statements

All procedures were carried out according to the guidelines on the ethical use of animals from the European community council directive (86/609/EEC) and were approved by the French minister of agriculture (OGM agreement 5080).

### Sanger sequencing of hemizygous P23H-1 rats

**Fig 1:**
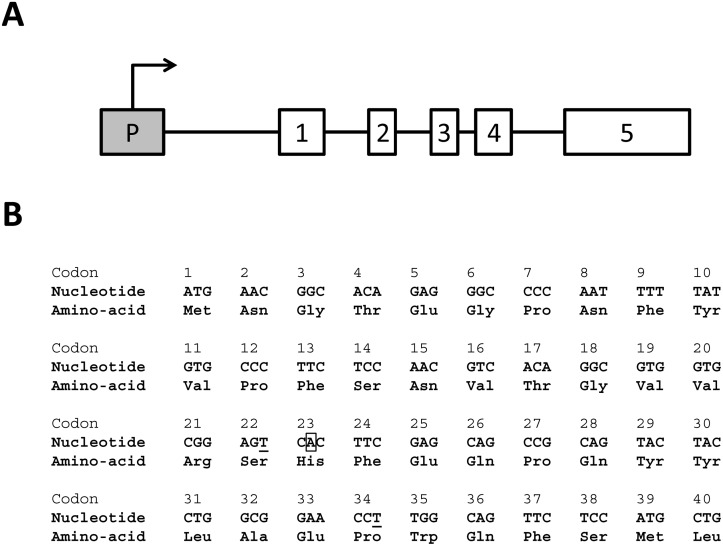
P23H mutated mouse *Rho* transgene. (A) Schematic drawing of the sequence: the transgene comprises the gDNA of mouse *Rho* from the promoter to the 5’UTR, encompassing the exons and introns. (B) Exon 1 first 40 codon sequence: the c.68C>A missense mutation leading to the p.Pro23His exchange is framed, and the 2 variations surrounding the mutation are underlined.

Genomic DNA (gDNA) was isolated from rat tails using a kit (DNeasy Blood & Tissue Kit, Qiagen, Venlo, The Netherlands). Nineteen couples of primers were designed to amplify the mouse *Rho* gene (NC_000072.6, gene structure depicted in Fig 1A) including the c.68C>A, p.Pro23His mutation (P23H mutated mouse *Rho* transgene) (Table 1). Amplicons were of 500-800-base-pair length covering the promoter region to the 3’ untranslated region (UTR) of the gDNA of mouse *Rho* in the P23H-1 rat. The following PCR parameters were applied: 15 min at 95°C for denaturation, 35 cycles of 45 sec at 95°C, 1 min at 58°C, and 30 sec at 72°C, and for final extension 10 min at 72°C (HOT FIREPol, Solis Biodyne, Tartu, Estonia). WT rat gDNA was used as control. When it was not possible to design probes to specifically amplify the P23H mutated mouse transgene, adjacent mouse-specific flanking primers were used to amplify larger genomic regions encompassing the fragment of interest (amplicon 6, 9, 10 and 17). The following PCR parameters were then applied: 15 min at 95°C for denaturation, 35 cycles of 45 sec at 95°C, 1 min at 58°C, and 1 min at 72°C, and for final extension 10 min at 72°C (HOT FIREPol). The specificity of PCR products was checked for correct size by electrophoresis on 2% agarose gels, subsequently Sanger sequenced with a sequencing mix (BigDyeTerm v1.1 CycleSeq kit, Applied Biosystems, Courtaboeuf, France) and primers (Table 1), on an automated 48-capillary sequencer (ABI 3730 Genetic analyzer, Applied Biosystems, Life technologies, Carlsbad, California, USA). Sequences were compared to the reference sequence (NC_000072.6) using appropriate software (SeqScape, Applied Biosystems).

**Table 1:** P23H mutated mouse transgene Sanger sequencing primers.

Primer	Sequence 5' to 3'
P23H_TG_1F	TAAACTGCTAGTGGCCAACTCC
P23H_TG_1R	GGATGCTCCAGGATGACTGT
P23H_TG_2F	GGTACTGGCTTCTTGCATCCA
P23H_TG_2R	GGGAGGAGACACATTCCTGT
P23H_TG_3F	CCCACATGCTCACCTGAATA
P23H_TG_3R	CATCTTGTCTGCCCCAGAGT
P23H_TG_4F	TCTGTCAAGTGAGCCATTGTC
P23H_TG_4R	GTACTGCGGCTGCTCGAAGT
P23H_TG_5F	GAGCCGTCAGTGGCTGAG
P23H_TG_5R	TGGGCCTTTAGATGAGACCA
P23H_TG_6F	GTGTAGCATGGGAGCCAAG
P23H_TG_6R	ACCTGGCGTAGCATAGTGGT
P23H_TG_7F	CGGCATCTCAAAGGATTCAT
P23H_TG_7R	GCATGGTACCCCAGCTTCTA
P23H_TG_8F	GGTAGCACTGTTGGGCATCT
P23H_TG_8R	GAGAGCAGGCTAGGATGCAA
P23H_TG_9F	AAGAGCTTCTGTTTTGGCACA
P23H_TG_9R	TTGGAATGTCCAGGGTTCTC
P23H_TG_10F	CGAAAACCATCCTGGTGACT
P23H_TG_10R	GATGAGGAAAGAGGCCAGTG
P23H_TG_11F	AGGCTGAACCTTCCCAAAAT
P23H_TG_11R	GCATGAATGGCTTTTACCTG
P23H_TG_12F	ACTCCCTTAACCACCGAAGG
P23H_TG_12R	CTAGCCCATGGCGTCTGTA
P23H_TG_13F	TGGTCCACTTCACCATTCCT
P23H_TG_13R	TTGGTCGGCTGTATCTCACA
P23H_TG_14F	GGAGGCATTGCACTCAGACT
P23H_TG_14R	CACACAGCTTAAATGGGACAGA
P23H_TG_15F	AAACGCCACAGTCTCTCTGC
P23H_TG_15R	TCAAGCTGTCCCCATTGAGT
P23H_TG_16F	AGATGACGACGCCTCTGC
P23H_TG_16R	CTGGATTTGGGAGATCCAAC
P23H_TG_17F	CATACCTGCCCTGGTTTTCT
P23H_TG_17R	CCACTTGGTTGCTGGTGTAG
P23H_TG_18F	AGATCCAGCCCTTCCTCTTG
P23H_TG_18R	ACTGCCTCAAATTGGGTTTC
P23H_TG_19F	TACACTTGGTGGCAGTGGTG
P23H_TG_19R	CCTTTCTGGAAGGGTGTCTG

### Transgene copy number quantification by quantitative PCR

**Fig 2:**
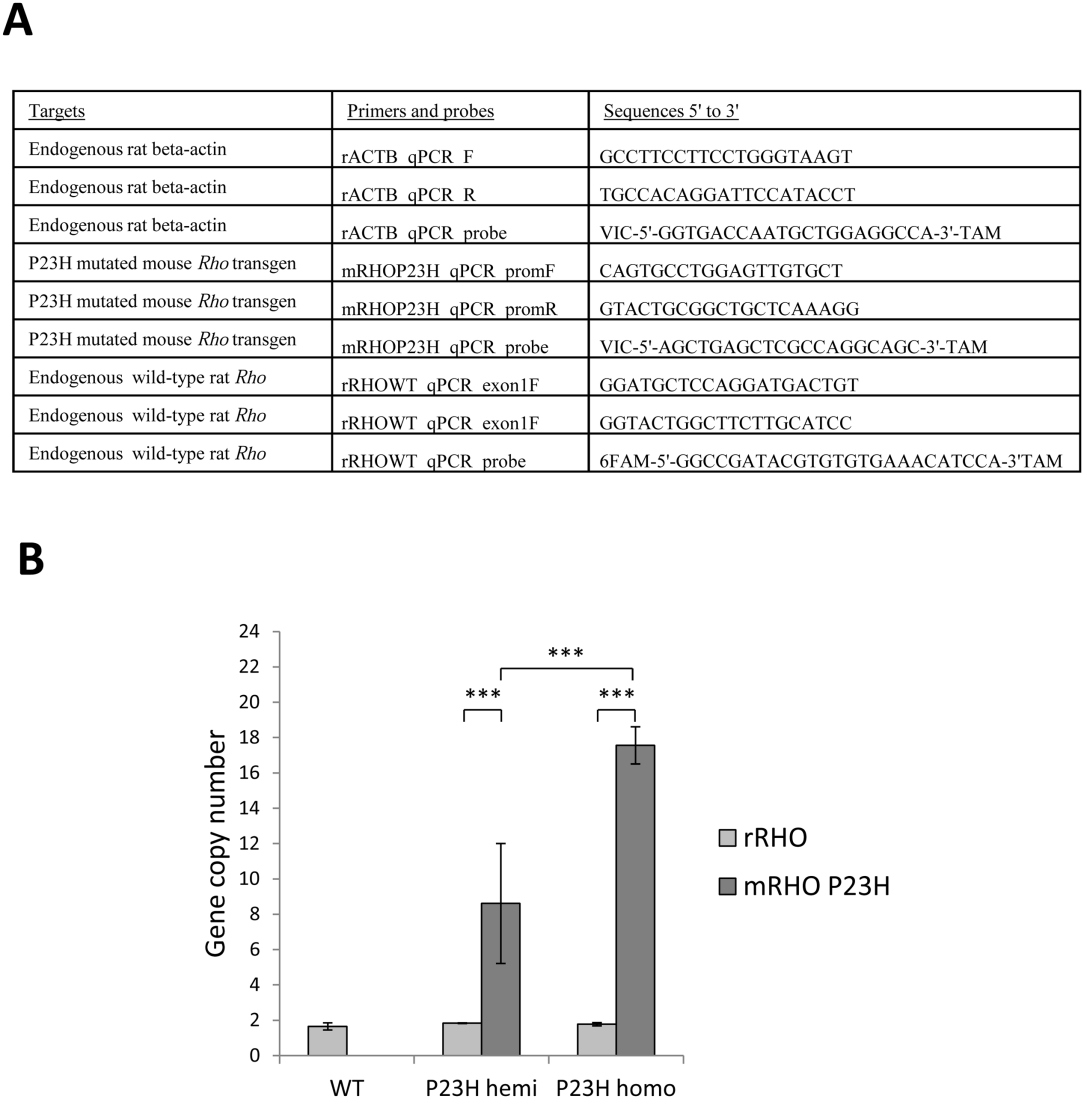
P23H mutated mouse *Rho* transgene copy number. (A) Target and sequence of primers and probes used for qPCR, FAM: 6-carboxyfluorescein and TAM: tetramethylrhodamine. (B) WT endogenous rat *Rho* (light grey) and P23H mutant mouse *Rho* (dark grey) copy numbers were evaluated by qPCR and 2^-ΔΔCt^ method for WT (WT), hemizygous P23H-1 (P23H hemi) and homozygous P23H-1 (P23H homo) rats. All experiments were conducted on the gDNA of 5 rats per group. Three asteriks (***) indicate a p value of <0.001, error bars represent standard errors.

Quantitative PCR (qPCR) to identify transgene copy number was adapted from Ballester and co-workers [21]. qPCR was conducted on 25ng of gDNA from 5 animals per genotype (WT, hemizygous P23H-1 and homozygous P23H-1 rats to check the validity of the method) with triplicate in a 20μl reaction using a kit (Taqman PCR Universal Master Mix no AmpErase UNG and the StepOnePlus Real-Time PCR system,both from Applied Biosystems). Probes (Applied Biosystems) were specific to the P23H mutated mouse transgene or the rat *Rho* gene (NC_005103.4). The final concentration of primers (Sigma-Aldrich, Saint-Louis, Missouri, USA) was 200 mM each and 250 nM for the probe (listed on Fig 2A). The qPCR was run using the following amplification parameters: 10 min at 95°C and 40 cycles of 15 sec at 95°C and 1 min at 60°C. Relative quantification of endogenous rat *Rho* and P23H mutated mouse *Rho* transgene was performed using the 2^-ΔΔCt^ method with the rat beta-actin gene for normalization.

### RNA extraction, reverse transcription and transgene expression measurement by quantitative PCR

**Fig 3:**
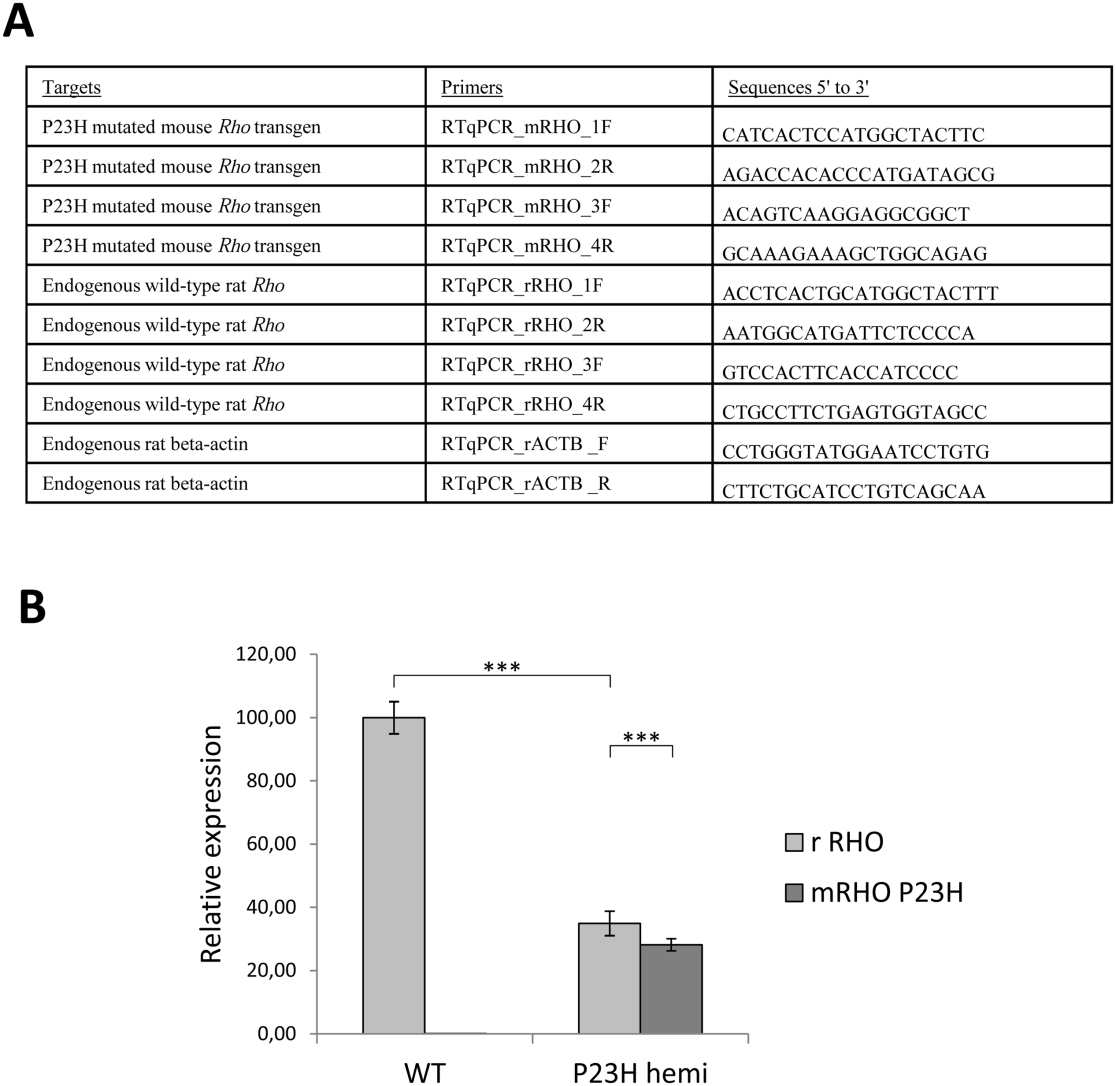
P23H mutated mouse *Rho* transgene expression. (A) Target and sequence of primers used for RT-qPCR. (B) WT endogenous rat *Rho* (light grey) and P23H mutant mouse *Rho* (dark grey) relative expressions were evaluated by qPCR and 2^-ΔΔCt^ method for 1-month-old WT (WT) and hemizygous P23H-1 (P23H hemi) rat retinas. In 1-month-old WT rats endogenous *Rho* expression was assessed at 100%. All experiments were conducted on the retinal cDNA of 5 rats per group. Three asteriks (***) indicate a p-value of <0.001, error bars represent standard errors.

Total RNA was isolated from 1-month-old rat retinas using a kit (RNeasy Mini Kit, Qiagen), followed by a complementary treatment with RNase-free DNase (Qiagen) to ensure the absence of gDNA. RNA integrity was verified by agarose gel electrophoresis. One microgram of total RNA was reverse-transcribed with oligo-dT using a transcriptase (Superscript II Reverse Transcriptase, Invitrogen, Life technologies, Carlsbad, California, USA) following the manufacturer's instructions. qPCR was conducted on cDNA of 5 animals per genotype (WT and hemizygous P23H-1 rats) using a kit (StepOnePlus Real-Time PCR system, Applied Biosystems) and 2 couples of specific primers for each P23H mutated mouse *Rho* transgene and endogenous rat *Rho*, and one couple of primers for rat beta-actin (Sigma-Aldrich, listed on Fig 3A). The equivalent of 1.25 ng of cDNA was used per well as template for qPCR reactions with a SYBR green master mix (Power SYBR green PCR Master Mix, Applied Biosystems). Each condition was performed in triplicates; *C*_t_ values were obtained with a software (ABI 7500,v.2.0.6, Applied Biosystems). Comparative ΔΔ*C*_t_ method with rat beta-actin as a housekeeping gene was used to determine the relative P23H mRNA from mouse *Rho* transgene *versus* endogenous rat *Rho*.

### Full field electroretinogram (ERG)

ERG was performed at age 1, 2, 3, 6 and 7 months on 12 WT and 14 hemizygous P23H-1 rats. After overnight dark adaptation, rats were anesthetized with ketamine (100 mg/kg) and xylazine (10 mg/kg). Eye drops were used to dilate the pupils (0.5% tropicamide) and anesthetize the cornea (0.4% oxybuprocaine chlorhydrate). Body temperature was maintained at 37°C using a circulating hot water heating pad. Upper and lower lids were retracted to keep the eyes open and bulging. Custom-made gold contact lens electrodes were placed on the corneal surface to record the ERG (VisioSytem, SIEM Bio-medicale, Nîmes, France). Needle electrodes placed subcutaneously in cheeks served as reference and a needle electrode placed in the back served as ground. Recordings were made from both eyes simultaneously. The light stimulus was provided by a Ganzfeld stimulator (Visiosystem, SIEM Bio-medicale). Responses were amplified and filtered (1 Hz-low and 300 Hz-high cut off filters) with a 1 channel DC-/AC-amplifier. Four levels of stimulus intensity ranging from 1.9 cd.s.m^-^² to 12 cd.s.m^-^² were used for the dark-adapted ERG recording. Each scotopic ERG response represents the average of 5 responses from a set of 5 flashes of stimulation. To isolate cone responses a 5-minute light adaptation at 20 cd.m^-2^ was used to saturate rod photoreceptors. A 12 cd.s.m^-^² level of stimulus intensity was used for the light-adapted ERGs. The light-adapted ERGs were recorded on the same rod-suppressive white background as for the light adaptation. Each cone photopic ERG response represents the average of 10 responses. *The major components of the ERG were measured conventionally* [22]. All comparisons were done at 12 cd.s.m^-^² of intensity (scotopic and photopic responses).

### Spectral domain optical coherence tomography (SD-OCT)

SD-OCT was performed at age 1, 2, 3 and 6 months on 9 WT and 9 hemizygous P23H-1 rats. Rats were anesthetized with ketamine (100 mg/kg) and xylazine (10 mg/kg). Eye drops were used to dilate the pupils (0.5% tropicamide) and eye dehydration was prevented by regular instillation of sodium chloride drops. SD-OCT images were recorded for both eyes using a spectral domain ophthalmic imaging system (Bioptigen, Inc., Durham, NC, USA). We performed rectangular scans consisting of a 2 mm by 2 mm perimeter with 1000 A-scans per B-scan with a total of 100 B-scans. Scans were obtained first while centering on the optic nerve, and then with the nerve displaced either temporally/nasally/dorsally or ventrally. SD-OCT scans were exported from InVivoVue as AVI files. These files were loaded into ImageJ (version 1.47; National Institutes of Health, Bethesda, MD, USA) where they were registered using the Stackreg plug-in. If the optic nerve was placed temporally/nasally, three B-scans at the level of the nerve were added, and measurements were performed 800 μm away from the optic disc, on each side. In the case where the optic nerve was placed dorsally/ventrally, 3 B-scans placed 800 μm away from the optic disc were added to perform the measurements. Thickness from the following layers were measured: ONL, outer plexiform layer (OPL), inner nuclear layer (INL), a complex comprising inner plexiform layer (IPL), ganglion cell layer (GCL) and nerve fiber layer (NFL) that we called IPL+GCL+NFL, and the whole inner retinal layers comprising OPL, INL and IPL + GCL + NFL

### Statistical analyses

Statistical analyses were performed using the Mann-Whitney U test on statistical software (SPSS, version 19.0 Inc, Chicago, Illinois, USA). For transgene copy number quantification by qPCR, each gene copy number in the same group, and copy number of the same gene in each group, were compared. For transgene expression quantification by RT-qPCR, each gene relative expression in the same group, and relative expression of the same gene in each group were compared. For scotopic ERG, a- and b-wave amplitudes and implicit times of maximum intensity (cd.s.m^-^²) stimulus responses were compared between WT and hemizygous P23H-1. For photopic ERG, a-waves were not analyzed because of the low amplitudes and high variability in recordings. However, b-wave amplitude and implicit time were compared between WT and hemizygous P23H-1 rats. To compare rod and cone dysfunction, differences between WT and hemizygous P23H-1 b-wave amplitude at each time were performed and compared between scotopic and photopic conditions with Wilcoxon signed-rank test. For SD-OCT, in each group, comparison of thickness for each layer at each time point between the 4 positions of acquisitions (temporal, nasal, dorsal, and ventral) revealed no statistical difference; therefore these values were combined. Then, all thickness values for each layer at a given time were compared between WT and hemizygous P23H-1 groups. Finally, Spearman rank correlation coefficients were calculated for P23H-1 rats on the means of scotopic a-wave amplitude for a stimulation of 12 cd.s.m^-^² and ONL thickness, scotopic a-wave and b-wave amplitudes for a stimulation of 12 cd.s.m^-^², and scotopic a-wave and b-wave implicit times for a stimulation of 12 cd.s.m^-^².

## Results

### P23H-1 rat genotyping

#### Transgene sequence

We first investigated the transgene sequence of the P23H-1 rat model thought to be a mouse P23H mutated allele inserted in a WT rat background. Sanger sequencing with mouse *Rho* specific primers revealed that this transgene contains the entire mouse opsin (*Rho*) genomic DNA (gDNA) from the promoter to the 3’UTR encompassing all exons and introns (Fig 1A). We confirmed the presence of the c.68C>A, p.Pro23His mutation. Two synonymous variations surrounding the P23H exchange (Fig 1B), were identified in addition to other variations in the promoter or 5’UTR (Table 2).

**Table 2:** P23H mutated mouse transgene variations.

Nomenclature	Type	Description
c.1-1448T>C	Upstream gene variant	rs31513717
c.1-1433A>G	Upstream gene variant	rs31513719
c.1-1290A>G	Upstream gene variant	rs31003645
c.1-1275A>G	Upstream gene variant	rs31513722
c.1-1201T>C	Upstream gene variant	rs31514804
c.1-1181A>G	Upstream gene variant	never described
c.1-1174dup	Upstream gene variant	never described
c.1-1139C>T	Upstream gene variant	rs31509484
c.1-1048A>G	Upstream gene variant	rs3666783
c.1-991G>A	Upstream gene variant	rs31509489
c.1-895T>A	Upstream gene variant	rs31509491
c.1-894del	Upstream gene variant	never described
c.1-700G>T	Upstream gene variant	rs30903457
c.1-546C>T	Upstream gene variant	never described
c.1-540dup	Upstream gene variant	never described
c.1-357A>G	Upstream gene variant	rs214120882
c.66C>T, p.Ser22Ser	Synonymous	never described
c.68C>A, p.Pro23His	Missense	Dryja, 1990, Nature
c.102A>T,p.Pro34Pro	synonymous	never described
c.1047+190G>A	3'UTR variant	rs31514859
c.1047+210T>C	3'UTR variant	rs31514861
c.1047+513T>C	3'UTR variant	rs21232304
c.1047+554A>G	3'UTR variant	rs237469398
c.1047+652T>G	3'UTR variant	rs250121950
c.1047+656A>G	3'UTR variant	rs115937375
c.1047+665_c.1047+675dup	3'UTR variant	rs262701018
c.1047+666A>G	3'UTR variant	rs250907798
c.1047+841C>T	3'UTR variant	rs239300315
c.1047+1249C>T	3'UTR variant	rs234137219
c.1047+1469C>T	3'UTR variant	rs31515781
c.1047+1531G>A	3'UTR variant	rs31515783
c.1047+1553A>G	3'UTR variant	rs216330615
c.1047+1591_c.1047+1592insT	3'UTR variant	rs217122073
c.1047+1629C>T	3'UTR variant	rs31516697
c.1047+2052G>A	3'UTR variant	rs107847221

#### Quantification of P23H transgene copy number

P23H mutated mouse transgene copy number was determined in both hemizygous and homozygous P23H-1 rats. Rat beta-actin normalization allowed us to determine the endogenous rat *Rho* copy number to be 2 copies for both hemizygous and homozygous P23H-1 rats, validating our method (Fig 2B). The P23H transgene copy number was statistically different in the different groups. Eighteen copies were counted for homozygous P23H-1 rats and 9 for hemizygous P23H-1 animals. As expected, no P23H mutated mouse transgene copy was found in WT rats (Fig 2B).

#### Quantification of P23H transgene expression

Endogenous rat *Rho* (*rRHO*) expression in 1-month-old WT rats was set at 100%. As expected, there was no P23H transgene expression in this group (Fig 3B). However, in 1- month-old hemizygous P23H-1 rats, endogenous wild-type *Rho* expression was reduced to 66% of age-matched WT (p<0.001). The mutated allele represented 43% of overall *Rho* expressed alleles, being also 20% lower than endogenous wild-type *Rho* (p<0.001).

### P23H-1 rat phenotyping

#### Full-field ERG recordings

**Fig 4:**
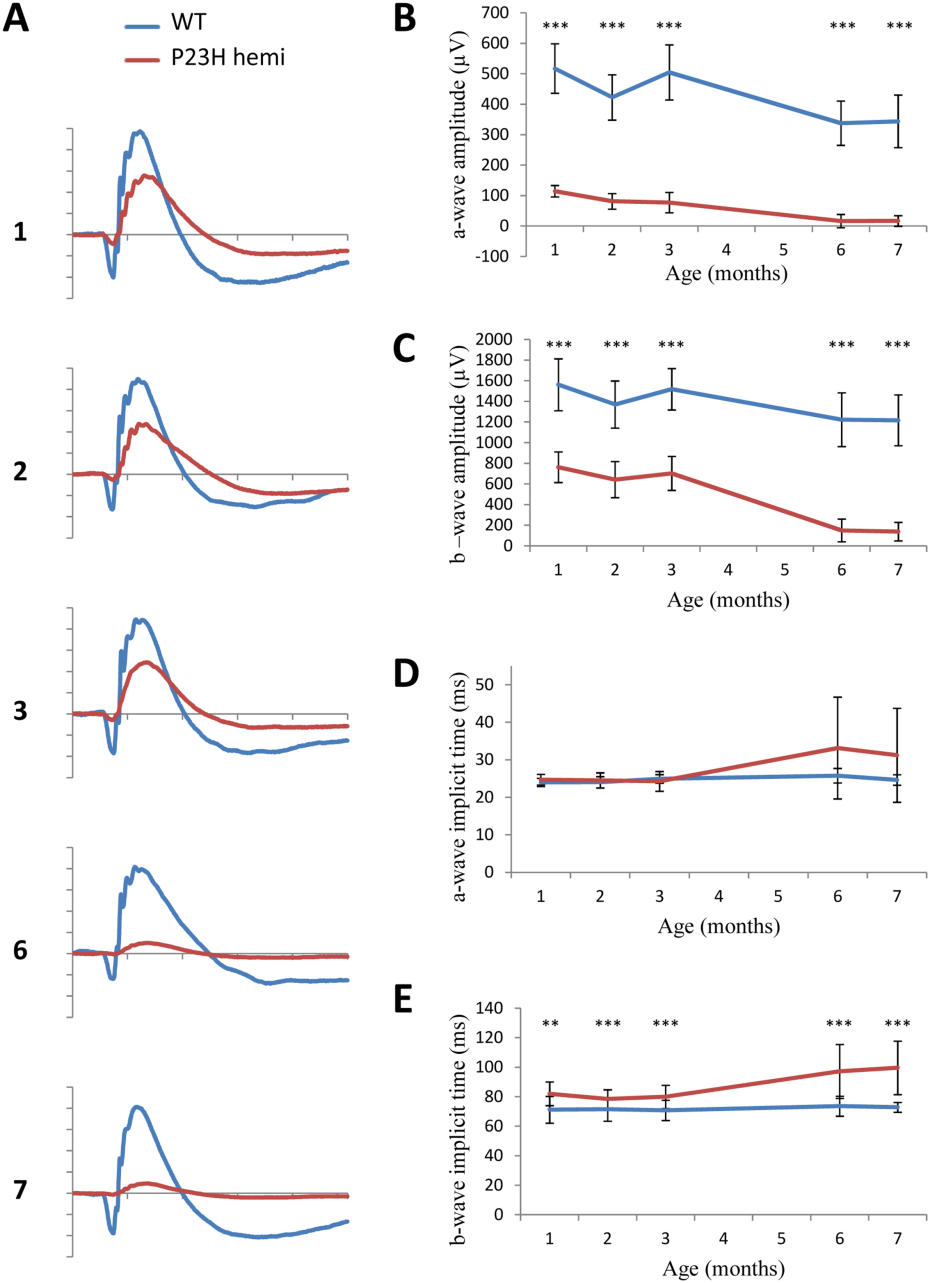
Scotopic ERG phenotype. Dark-adapted ERG series were obtained from representative WT (n = 12, blue line) and hemizygous P23H-1 (n = 14, red line) rats at 1,2,3,6 and 7 months of age and a 12 cd.s.m^-^² stimulus intensity. (A) Representative waveforms, as a function of age. The scale marks indicate 50ms (time in abscissa) and 300μV (amplitude in ordinate) (B-C) Mean maximum amplitudes of a-wave (B) and b-wave (C) with increasing age. (D-E) Mean implicit times of a-wave (D) and b-wave (E) with increasing ages of rats. Asteriks indicate a significant test (** for p<0.005 and *** for p<0.001) between WT and hemizygous P23H-1 responses, error bars represent standard errors.

ERG responses of WT and hemizygous P23H-1 rats were recorded at 1-, 2-, 3-, 6- and 7-months of age under scotopic and photopic conditions, allowing us to estimate respectively rod and cone photoreceptor function. Results are displayed for 12 cd.s.m^-^² stimulus intensity. Under scotopic conditions, amplitudes of both a- and b-waves were reduced as early as 1 month after birth in hemizygous P23H-1 rats compared to WT rats (p<0.001 at each age). This decrease is accentuated with age leading to a flat a-wave at 6 months and an 88% reduction in b-wave amplitude at 7 months (137μV for hemizygous P23H-1 rats and 1216 μV for WT rats, Fig 4A–4C). Moreover, b-wave implicit time is increased starting at 1 month of age (p<0.01), with a maximum of 35% increase reached at 7 months (p<0.001, Fig 4A and 4E). In contrast, there is no significant change in implicit time of a-wave (Fig 4A and 4D). Taken together, these results indicate rod photoreceptor dysfunction progressing with age.

**Fig 5:**
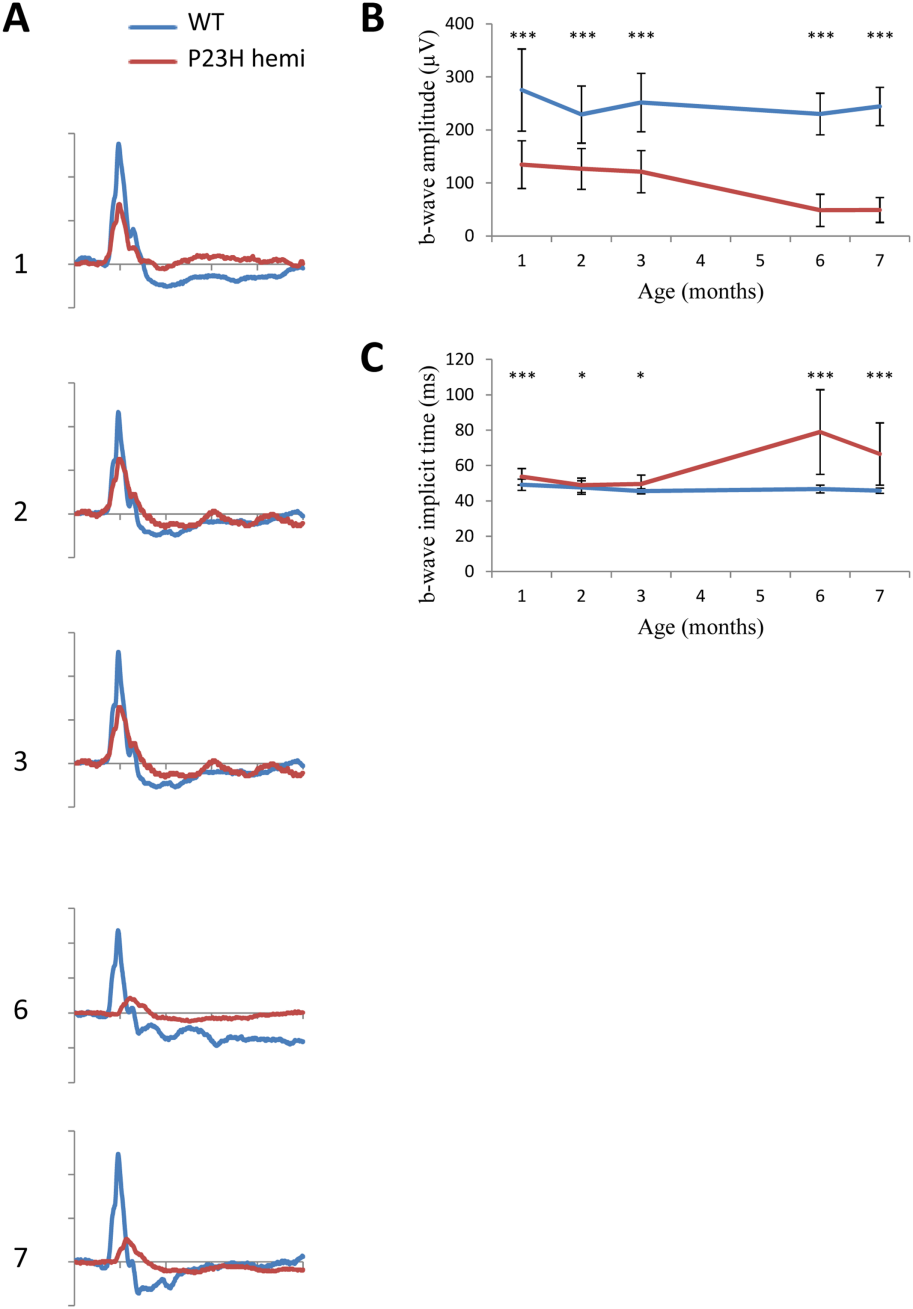
Photopic ERG phenotype. Light-adapted ERG series were obtained from representative WT (n = 12, blue line) and hemizygous P23H-1 (n = 14, red line) rats at 1,2,3,6 and 7 month of age and at 12 cd.s.m^-^² stimulus intensity after a 5-minute light adaptation at 20 cd.m^-^². (A) Representative waveforms, as a function of age. The scale marks indicate 50ms (time in abscissa) and 100μV (amplitude in ordinate) (B-C) Mean maximum amplitudes (B) and implicit times (C) of b-wave in time. Because of the limit of sensitivity of the method, the a-wave component is not plotted. Asteriks indicate a significant test (** for p<0.005 and *** for p<0.001) between WT and hemizygous P23H-1 rats’ responses, error bar represents standard errors.

In photopic conditions, a-wave amplitudes and implicit times are difficult to reliably measure due to their small amplitudes both in WT and mutant rats. Nevertheless, b-wave amplitudes and implicit times are well defined. As observed under scotopic conditions, b-wave amplitude is reduced from the first month of age in mutant compared to WT animals (p<0.001 at each age), and this decrease progresses with age reaching 78% of diminution between hemizygous P23H-1 and WT rats at 7 months of age. Implicit time also increases starting at 1 month with a maximum increase of 46% reached at 7 months of age (Fig 5A–5C). When comparing a- and b-wave amplitude, the Spearman rank correlation coefficient was ρ = 0.87 (p<0.0001) on WT animals and ρ = 0.83 (p<0.0001) on P23H-1 hemizygous rats while comparing the a- and b- wave implicit time ρ = 0.47 (p = 0.00261) for WT animals and ρ = 0.627 (p<0.0001) for P23H-1 hemizygous rats.

#### Retinal layers assessment of the P23H-1 rats by SD-OCT

**Fig 6:**
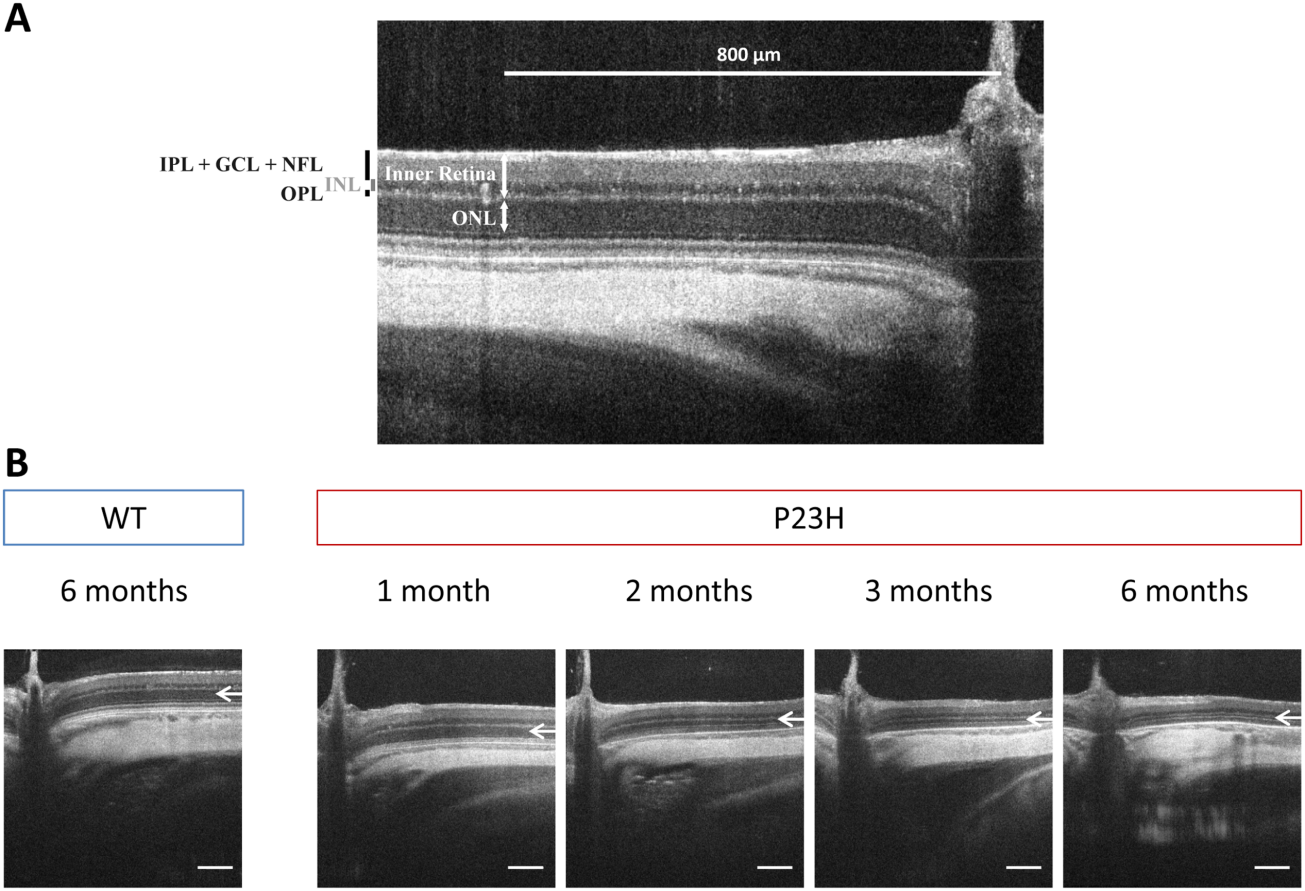
SD-OCT retinal thickness layers and retinal morphology (A) Outer nuclear layer (ONL), outer plexiform layer (OPL), inner nuclear layer (INL), a complex comprising inner plexiform layer (IPL), ganglion cell layer (GCL) and nerve fiber layer (NFL) called IPL+GCL+NFL, and inner retinal layer thickness are measured at 800 μm of the optic nerve on SD-OCT images (B) Representative SD-OCT section of a 6-month-old WT rat and 1-, 2-, 3-, and 6-month-old hemizygous P23H-1 rats. White arrows depict ONL. Scale bar: 200μm.

**Fig 7:**
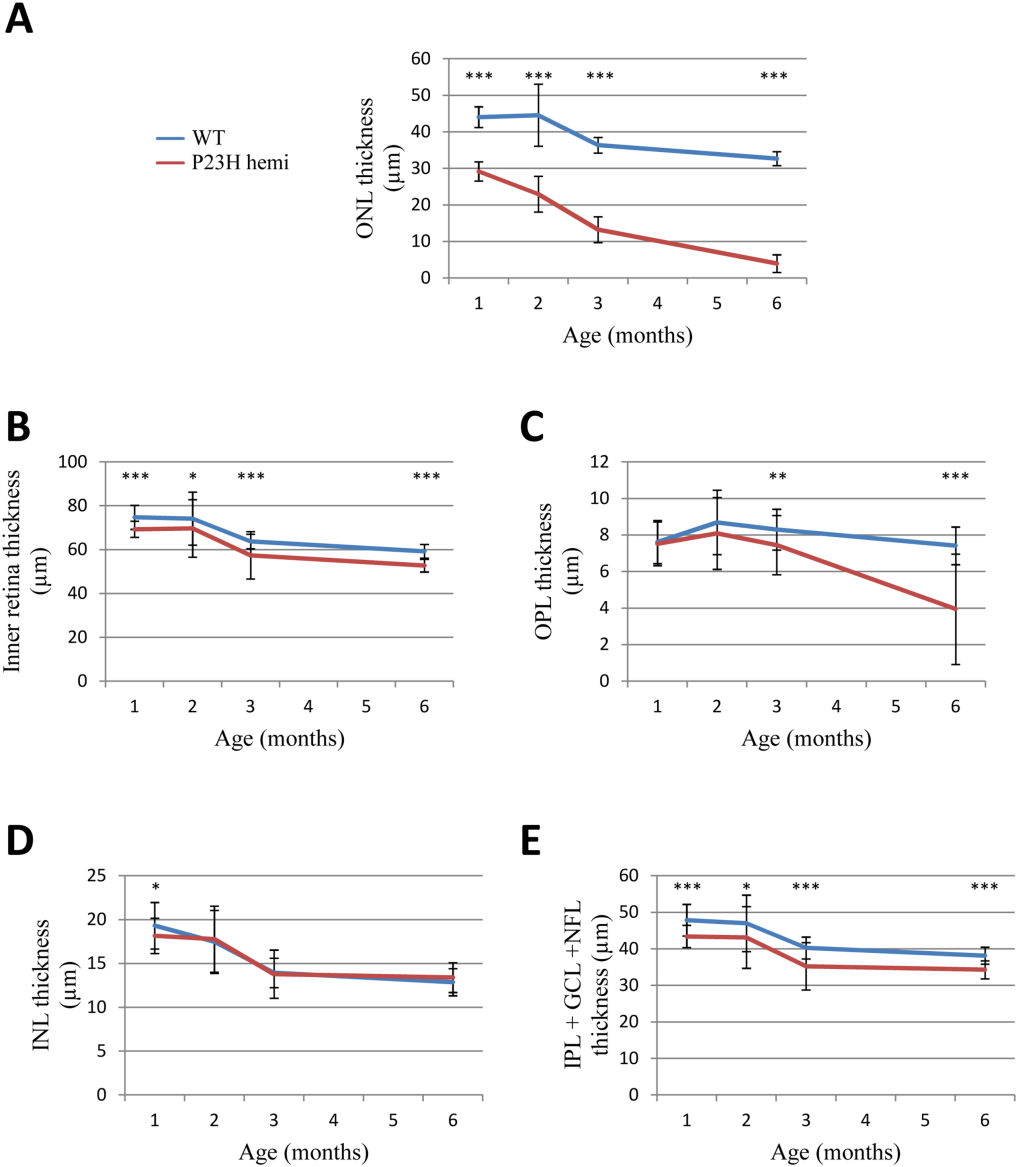
SD-OCT retinal thickness layer monitoring. Retinal layer thicknesses are measured at 800 μm of the optic nerve on SD-OCT images and compared to WT (blue line) and hemizygous P23H-1 (red line) at 1, 2, 3 and 6 months of age. (A) Outer nuclear layer (ONL), (B) inner retinal layer, (C) outer plexiform layer (OPL), (D) inner nuclear layer (INL), (E) a complex comprising inner plexiform layer (IPL), ganglion cell layer (GCL) and nerve fiber layer (NFL) called IPL+GCL+NFL. Asteriks indicate the following p values: * for p<0.05 and *** for p<0.001, error bars represent standard errors.

Representative SD-OCT images from 1-, 2-, 3- and 6-month hemizygous P23H-1 rats, compared to WT retinas, show a progressive thinning of the ONL (composed of rod and cone photoreceptor nuclei, described in Fig 6A and depicted by white arrows in Fig 6B). WT and hemizygous P23H-1 rat retinal layers (ONL, OPL, INL, IPL+GCL+NFL and inner retinal layers, described in Fig 6A) are all thinned between 1 and 6 months of age (Fig 7 and Table 3). When studied separately, some changes appear between WT and hemizygous P23H-1 rat retinal layers. Hemizygous P23H-1 rat ONL thins with aging to finally reach 86% of decrease at 6-months-of-age when WT rat ONL is reduced by 27% (p<0.001, Fig 7A and Table 3). One-month hemizygous P23H-1 rat inner retina is thinner than WT, and this difference is maintained but not modified with aging (Fig 7B and Table 3). If layers composing the inner retina are studied separately, changes appear in the OPL. Hemizygous P23H-1 rat OPL are decreased at 3-months (p<0.005) and 6-months (p<0.001) of age when compared to WT (Fig 7C and Table 3). Inner nuclear layer from 1-month hemizygous P23H-1 rat is thinner than WT (p<0.05), but this difference disappears with aging (Fig 7D and Table 3). As observed for inner retina, 1-month hemizygous P23H-1 rat IPL + GCL + NFL is thinner than WT, and this difference remains constant with aging (Fig 7E and Table 3).

**Table 3:** Retinal layer thickness measured by SD-OCT in WT and hemizygous P23H-1 rats at 1, 2, 3 and 6 months of age.

	WT				P23H-1			
	1 month	2 months	3 months	6 months	1 month	2 months	3 months	6 months
ONL	44μm ±3μm	43 μm ±11 μm	36 μm ±2 μm	33 μm ±2 μm	29 μm ±3 μm	23μm ±5 μm	13 μm ±3 μm	3 μm ±2 μm
OPL	8μm ±1 μm	9 μm ±2 μm	7 μm ±3 μm	7 μm ±1 μm	8 μm ±1 μm	8 μm ±2 μm	8 μm ±1 μm	7 μm ±3 μm
INL	19 μm ±3 μm	17 μm ±4 μm	12 μm ±5 μm	13 μm ±2 μm	18 μm ±2 μm	18 μm ±4 μm	14 μm ±2 μm	13 μm ±3 μm
IPL+GCL+NFL	48 μm ±4 μm	47 μm ±4 μm	36 μm ±13 μm	38 μm ±2 μm	43 μm ±3 μm	43 μm ±6 μm	36 μm ±2 μm	34 μm ±2 μm
Inner retinal layers	75 μm ±5 μm	74 μm ±12μm	57 μm ±20 μm	60 μm ±3 μm	69 μm ±3 μm	70 μm ±13 μm	59 μm ±3 μm	53 μm ±3 μm

(mean ± standard deviation; ONL: outer nuclear layer, OPL: outer plexiform layer, INL: inner nuclear layer, IPL: inner plexiform layer, GCL: ganglion cell layer, NFL: nerve fiber layer)

## Discussion

The present study reports the exact sequence, copy number and retinal expression level of the P23H mutated transgene in the P23H-1 rat model, using Sanger sequencing, qPCR and RT-qPCR analysis. Furthermore it documents in greater detail the disease course in this rat model with functional and structural phenotypic assessment using *in vivo* evaluation by ERG and SD-OCT.

P23H rat models were developed for translational research and study photoreceptor rescue strategies [23, 24]. By reproducing the degeneration observed in patients [19], these animal models are extremely helpful for therapeutic approaches for adRP, a yet incurable disease. P23H-1 rat model is the most commonly used model since it carries the most frequent mutation in adRP in the US [1] and displays relatively fast degeneration over 6 months (http://www.ucsfeye.net/mlavailRDratmodels.shtml). This allows an easy documentation of the phenotype and fast monitoring of putative therapeutic rescue. Moreover, hemizygous animals were preferred to homozygous since it was supposed to more closely resemble the human autosomal dominant genetic conditions with one mutated allele and present a slower degeneration, more suitable for translational research. Several strategies of photoreceptor [25] and Muller glial cell [26] transplantation or administration of anti-apoptotic [27, 28], heat shock response activator [29] or enhancer [30], anti-aggregating [31], or neuroprotective [32, 33] molecules and chaperones [34] have been attempted to slow the degeneration and demonstrated partial preservation of photoreceptor morphology and function. However, treatment efficacy was evaluated in comparing treated and untreated animals less than 1 month after treatment, or after 6 months of age, a time point at which photoreceptor degeneration is almost complete. RNA-targeted therapy by gene silencing using ribozymes directed against the mouse transgene in P23H Line 3 rat model has also been attempted [23, 24, 35] and revealed some preservation of the ONL thickness. Functional characterization by ERG shows a slight increase in response amplitude in treated *versus* untreated eyes after 3 months of age [23, 35]. These approaches indicate that genetic-targeted therapies including selective mutated allele suppression or gene replacement at the DNA or RNA level could be tested. Despite multiple studies that attempt therapeutic intervention in the P23H model, the genotype of this animal model was not yet fully available. We first addressed this question by characterizing the exact sequence of the mutated transgene in P23H-1 rats. Our data confirmed that the transgene contains the entire mouse *Rho* genomic DNA sequence including the promoter, all 5 exons and 4 introns with the P23H mutation as well as most of the 3’UTR. Variations were also identified in the promoter, exon 1 and 3’UTR. These include [c.66C>T, p.Ser22Ser] and [c.102A>T, p.Pro34Pro] (Fig 1B). These variations are silent and most likely predicted to be benign but will need to be taken into account while investigating genome editing strategies. Furthermore, we investigated transgene copy numbers and identified that homozygous and hemizygous P23H-1 rats carry 18 and 9 copies of the P23H transgene, respectively. This is coherent with the Mendelian transmission of this transgene with half of the copies of the homozygous in the hemizygous P23H-1 rats supporting a tandem insertion at a unique locus. This result led us to investigate if all transgene copies were expressed. In 1-month-old hemizygous P23H-1 rat retinas, WT *Rho* expression was 66% reduced compared to the age-matched WT rats. This result was consistent with photoreceptor degeneration due to retinitis pigmentosa [31]. Interestingly, in 1-month-old hemizygous P23H-1 rats, the mutated allele represented 43% of overall *Rho* expressed alleles, despite the 9 copies of the transgene. This resembles what is expected in patients with the P23H mutation, where both normal and mutated alleles are thought to be expressed at the same levels. This low expression of transgene in hemizygous P23H-1 rats could be due to WT and P23H *Rho* expression regulation at the mRNA levels, as described in a P23H mouse line with genomic mutated mouse opsin transgene [36]. Intrinsic silencing effect of multiple-copy tandem repeats on transgene expression could also be an explanation, since transcription inhibition can occur when more than 5 copies of the transgene are present [37, 38]. Insertion site of the transgene is also important since chromatin structure influences the expression level [39]. This result is favorable for intervention applying gene therapy, since low level of transcript is more likely to respond to gene silencing at the mRNA level than an overexpressed transgene. Altogether, our study provides important details on the P23H-1 rat’s genotype by describing the exact transgene sequence with its P23H mutation and variations, its copy number and expression. These data can serve basis for mutated allele specific therapeutic interventions using this model. Nevertheless, we cannot exclude a drift of the inserted copy number in the various colonies of P23H rat available to the scientific community and would recommend testing such parameter before attempting therapeutic studies especially those aiming at genome editing.

Another purpose of this work was to better monitor natural history of the disease applying functional and high-resolution *in vivo* phenotyping techniques. Functional characterization of hemizygous P23H-1 rats revealed reduced rod and cone response amplitudes and delayed implicit time as seen in RP patients [1, 3, 40]. In both scotopic and photopic conditions, hemizygous P23H-1 rats already displayed decreased responses at 1-month, enhanced by aging especially after 3-months and flatting at 6-months of age. Scotopic responses were more affected than photopic responses, indicating that rod photoreceptor dysfunction was more severe than cone photoreceptor dysfunction. These results are concordant with previous observations [17] and confirmed the usefulness of this model to mimic human disease with rod-cone dystrophy [1, 3, 40, 41]. Our study also provides retinal thickness monitoring using high resolution imaging with SD-OCT in hemizygous P23H-1 rats. Indeed, this non invasive *in vivo* technique enables longitudinal studies and now achieves the resolution of histology, without its terminal nature. Moreover, this alleviates the fixation process on retinal sections, which is a potential source of variation in structure and thickness measurements [42]. Such structural studies applying SD-OCT may provide surrogate markers to monitor functional rescue in translational research. When observed with aging in our study, structural characterization of hemizygous P23H-1 rats revealed gradual ONL thinning reaching 86% at 6-months of age, consistent with histology [17]. Of note, 1- and 2-month-old P23H-1 rats showed slightly different ONL thickness compared to the histological data (respectively 20μm and 10μm) from the literature (http://www.ucsfeye.net/mlavailRDratmodels.shtml). Difference between SD-OCT and histology measurements were also described on the same animals, but good correlation in the decreasing values makes this technique interesting for monitoring [41]. Moreover, while the degeneration seems to be slowed herein, ONL thicknesses reached comparable values. Animal housing conditions such as light intensity and feeding were different between this and other studies, and may also explain an initially slower degeneration documented in the present study [43–46]. In a recent study, SD-OCT and retinal thickness measurements on histology were assessed in 4-months old pigmented hemizygous P23H rats [41]. These animals were obtained by crossing homozygous P23H-1 with Long Evans rats to more closely resemble the human disease providing that albino rodents are more sensitive to photic trauma [47]. The 4-months old pigmented hemizygous P23H-1 and WT rats showed less degeneration than the 3-months old albino hemizygous P23H-1 and WT albino rats in the present study and their pigmented status could account for this difference. However, the difference in genetic background brought by crossing may also induce variations in the course of the disease [48, 49]. Furthermore, patients with a P23H mutation show regional distribution of retinal degeneration [50, 51]. In the P23H mouse, if dark reared, photoreceptor cell death was uniformly distributed across the retina, but when raised in cyclic light conditions, the density of apoptotic cells was greater, particularly in the inferior region of the retina [52], the region most severely affected in patients with the P23H mutation [50]. Photoreceptor degeneration was also accelerated by light exposure. Regional differences were not observed in our study on hemizygous P23H-1 nor on pigmented hemizygous P23H-1 rats [41] and one reason may be artificial light conditions in which they were raised. Interestingly, our study also demonstrates inner retinal abnormalities with no change in overall inner retinal thickness with age. Moreover, inner retinal abnormalities were described by OCT in regions where the ONL was thinned in patients with the P23H mutation [53]. Here we showed that hemizygous P23H-1 rats OPL thickness decreased dramatically with aging. This result is consistent with observations obtained on synaptic complexes by immunohistochemistry, where dendrites of bipolar cells were found less profuse in hemizygous albino and pigmented P23H-1 compared to WT, especially with aging [18, 41]. Synaptic connectivity of horizontal cells also changed after 3-months of age, with their dendrites being atrophied and condensed [41]. INL thickness composed of nuclei of bipolar, horizontal and amacrine cells, showed an initial decrease of 1μm in hemizygous P23H-1 compared to WT rats. With aging, no other difference appeared in the thickness of both hemizygous P23H-1 and WT rat INL. Immunohistochemistry on hemizygous albino P23H-1 rats showed that bipolar cell bodies were smaller at 40 days of age and confined to a single layer adjacent to the OPL and decrease in number between 5- and 9-months of age [18]. On pigmented hemizygous P23H-1 rats cell bodies were smaller after 2-months of age for horizontal cells and rod and cones bipolar cells, with a reduction in number particularly after 6-months of age [41]. These differences were not compared to the age-matched pigmented WT rats. We observed similar decreases by SD-OCT on albino WT and hemizygous P23H-1 rats, the role of the albino background on this degeneration remains also unclear. IPL+GCL+NFL thickness, comprising ganglion cells, their synapses with INL cells, and their axons forming the optic nerve, was 10% thinner in hemizygous P23H-1 than in WT rats. These results were consistent with immunohistochemistry on albino hemizygous P23H-1 rats, showing that ganglion cell population was initially smaller in hemizygous P23H-1 than in WT rats, and a difference in degeneration appeared after 6 months of age [19]. Immunohistochemistry on pigmented hemizygous P23H-1 rats showed horizontal and rod bipolar cells axons and varicosities were reduced after 2-months of age but these differences were not compared to the matched-aged pigmented WT rats [18, 41]. Finally, while correlating outer and inner retinal function (i.e., a- and b-wave parameter correlation), we find a close correlation that may imply the absence of additional inner retinal dysfunction to photoreceptor dysfunction suggesting that structural abnormalities do not result in functional alteration. However, the abnormalities observed in the inner retina need to be taken into account in the development of therapies aiming at restoring photoreceptor function, such as photoreceptor transplantation [54], subretinal implants [55], and optogenetic activation of dormant cone photoreceptor circuitry [56], as they all need functional inner retinal cells to be effective. A precise assessment would therefore be helpful to select candidates for appropriate treatments such as implants that could be placed either sub or epi-retinally and optogenetics that could be implemented on cones [56], bipolar [57] or ganglion cells [58] depending of these findings.

To conclude, this study clarifies the genotype of hemizygous P23H-1 rats, by identifying transgene sequence, numbering the gene copies and their expression. Functional and structural phenotypic and non-invasive *in vivo* evaluation by ERG and SD-OCT confirm previously reported functional and histological data in the literature and provide new endpoints for validation of preclinical therapeutic intervention. These results will serve as basis for the development of novel therapies, in particular those directly targeting the gene defect.
